# A Health Impact Assessment of a Proposed Bill to Decrease Speed Limits on Local Roads in Massachusetts (U.S.A.)

**DOI:** 10.3390/ijerph111010269

**Published:** 2014-10-02

**Authors:** Peter James, Kate Ito, Rachel F. Banay, Jonathan J. Buonocore, Benjamin Wood, Mariana C. Arcaya

**Affiliations:** 1 Department of Environmental Health, Harvard School of Public Health, 401 Park Drive, Boston, MA 02215, USA; E-Mail: rachel.banay@mail.harvard.edu; 2 Department of Epidemiology, Harvard School of Public Health, 401 Park Drive, Boston, MA 02215, USA; 3 Metropolitan Area Planning Council, 60 Temple Place, Boston, MA 02111, USA; E-Mails: kito@mapc.org (K.I.); rbanay@mapc.org (R.F.B.); marcaya@mapc.org (M.C.A.); 4 Center for Health and the Global Environment, Harvard School of Public Health, 401 Park Drive, Boston, MA 02215, USA; E-Mail: jbuonocore@mail.harvard.edu; 5 Massachusetts Department of Public Health, Division of Prevention and Wellness, 250 Washington Street, Boston, MA 02108, USA; E-Mail: ben.wood@state.ma.us; 6 Harvard Center for Population and Development Studies, 9 Bow Street, Cambridge, MA 02138, USA; E-Mail: mca767@mail.harvard.edu

**Keywords:** health impact assessment, speed limits, crashes, injury prevention, air pollution, physical activity, monetization

## Abstract

Decreasing traffic speeds increases the amount of time drivers have to react to road hazards, potentially averting collisions, and makes crashes that do happen less severe. Boston’s regional planning agency, the Metropolitan Area Planning Council (MAPC), in partnership with the Massachusetts Department of Public Health (MDPH), conducted a Health Impact Assessment (HIA) that examined the potential health impacts of a proposed bill in the state legislature to lower the default speed limits on local roads from 30 miles per hour (mph) to 25 mph. The aim was to reduce vehicle speeds on local roads to a limit that is safer for pedestrians, cyclists, and children. The passage of this proposed legislation could have had far-reaching and potentially important public health impacts. Lower default speed limits may prevent around 18 fatalities and 1200 serious injuries to motorists, cyclists and pedestrians each year, as well as promote active transportation by making local roads feel more hospitable to cyclists and pedestrians. While a lower speed limit would increase congestion and slightly worsen air quality, the benefits outweigh the costs from both a health and economic perspective and would save the state approximately $62 million annually from prevented fatalities and injuries.

## 1. Introduction

Motor vehicle crashes are the top cause of death among people aged 5 to 34 in the United States, and a leading cause of injury among all age groups [1]. Decreasing traffic speeds increases the amount of time drivers have to react to road hazards, potentially averting collisions, and makes crashes that do happen less severe [2]. Consistent evidence over the past century has confirmed that lowering traffic speeds decreases the frequency of crashes, as well as rates of fatalities and injuries due to vehicle collisions. This holds true on urban and residential roads [3,4]. This impacts both individuals traveling in vehicles, as well as pedestrians and cyclists who often share roadways with vehicles.

In 2012, the Massachusetts State Legislature considered a bill that would lower the default speed limit on “functionally classified local roads” from 30 miles per hour (mph) to 25 mph. U.S. Department of Transportation’s Federal Highway Administration uses functional classifications to group streets and highways into ‘classes’. Functional classification defines the role a road or street should play in serving mobility or access [5]. There are three highway functional classifications: arterial, which provides mobility at the greatest speed for the longest uninterrupted distance; collector, which provides service at a lower speed for shorter distances by collecting traffic from local roads and connecting them with arterials; and local, which primarily provides access to land with little or no mobility [5].

The aim of the proposed legislation was to reduce vehicle speeds on local roads across the state to a level that is safer for pedestrians, cyclists, and children. The legislation also allowed for municipalities to officially lower speed limits on their roads, which is currently difficult for many cities and towns in Massachusetts. The passage of the proposed legislation had potential far-reaching and important public health impacts. For example, lower default speed limits have the potential to affect the number of fatalities and serious injuries to motorists, cyclists and pedestrians; promote active transportation by making local roads feel more hospitable to cyclists and pedestrians; and change the concentration and composition of both near-roadway and regional pollutants, thereby potentially affecting cardiovascular and respiratory health across the Commonwealth of Massachusetts.

Boston’s regional planning agency, the Metropolitan Area Planning Council (MAPC), conducted a Health Impact Assessment (HIA) on the potential health impacts of the proposed bill in coordination with the Massachusetts Department of Public Health (MDPH), Central Transportation Planning Staff (CTPS), and stakeholders in the Commonwealth of Massachusetts. To assess how changes to speed limits on local roads might impact health, MAPC reviewed transportation-related literature to estimate how lowering speed limits would affect traffic speeds across the state; worked with CTPS to build statewide models that estimated the impact of new traffic speeds on vehicle miles traveled, vehicle hours traveled, and air quality in the region; and applied findings from peer-reviewed public health literature to the results of the CTPS transportation models to predict likely health outcomes, in consultation with local experts in the fields of transportation safety, environmental health, and active transportation.

## 2. Methods

### 2.1. Decision-Makers and the Decision-Making Process

Massachusetts State Representative Denise Provost of Somerville, working with state agencies, regional planning agencies, and advocacy organizations, filed House Bill 1808, *An Act relative to speed limits*, (hereafter referred to as the Speed Limit Bill) at the beginning of the 2011–2012 legislative session. Based on conversations with Representative Provost, an analysis of the potential health impact of the legislation was proposed to help legislators and their constituents develop more comprehensive and informed positions on the issue. This project was funded by MDPH’s Healthy Community Design through Health Impact Assessment Grant in spring of 2012. The time-sensitive nature of the Speed Limit Bill did not allow for an extensive review and modeling of health impacts. Therefore, the assessment presented in this report is from a “rapid” HIA and the analysis was completed in a short time frame.

### 2.2. Stakeholder Engagement

Stakeholder engagement ensures that an HIA is a transparent tool. The stakeholder engagement strategy for this rapid HIA focused largely on decision makers, transportation experts, and advocacy groups. MAPC and MDPH engaged stakeholders through an HIA training in June 2012. The goal of this training was to gather cross-sector stakeholders, introduce the goals and steps of HIA, and provide an overview of the Speed Limit HIA specifically. Eight stakeholders attended the HIA training in Boston, including Massachusetts State Representatives, bicycle and pedestrian advocates, representatives from the City of Boston, and transportation and air pollution specialists. To follow up with stakeholders after the training, we distributed a draft scope of the HIA for comment, including a diagram that outlined pathways between the Speed Limit Bill and health. Stakeholders also reviewed draft recommendations and provided comments later in the process.

Prior to the release of the HIA, MAPC held a “Slow Down Summit” in January 2013 to convene stakeholders and share the preliminary findings of the HIA. The Summit provided an opportunity to disseminate preliminary findings, as well as allowed stakeholders to submit feedback to the HIA process.

In addition to the cross-sector stakeholder engagement as part of the HIA training, MAPC leveraged its status as one of the thirteen regional planning agencies (RPAs) in Massachusetts to incorporate additional perspectives in the scoping process. We presented our draft scope and methodology to the executive directors of the thirteen RPAs at a monthly Massachusetts Association of Regional Planning Agencies (MARPA) meeting. Our goal was to make the executive directors representing the other regions of the state aware of the project and allow them an opportunity to provide feedback, and to ask them to support our request for each RPA’s transportation planners to vet a transportation modeling methodology to estimate the impact of the Speed Limit Bill on traffic patterns across the state (See Traffic Modeling). With support from all 13 RPA directors, we solicited and incorporated feedback on our modeling approach from transportation planners across the state.

### 2.3. Traffic Modeling

To assess the potential health impacts of the Speed Limit Bill, we first had to understand the effects of the bill on transportation patterns. MAPC subcontracted with the Central Transportation Planning Staff (CTPS), a multimodal transportation planning and analysis agency for Eastern Massachusetts, to model the statewide transportation impact of reducing speed limits on functionally classified local roads from 30 mph to 25 mph. This model predicted how changes in speed limits would likely affect average traffic speeds, mode shares (i.e., the percentage of trips taken by car, transit, and other modes), vehicle miles traveled, vehicle hours traveled, and air pollution emissions.

Previous research suggested that new speed limits would slow traffic, but not by the full 5 mph proposed by the bill (i.e., there would not be full compliance with the reduced speed limit). Based on data from multiple traffic studies, Elvik [2] found a non-linear relationship between changes in speed limits and subsequent changes in average traffic speeds. Specifically, Elvik’s analysis predicts that a 5 mph decrease in the speed limit would translate to a 1.8 mph decrease in average traffic speeds under free flow conditions where the speed limit, rather than congestion, determines vehicle speeds.

CTPS then modeled the impact of a 1.8 mph decrease on local roads under free flow conditions on 24 h averages, which incorporated congestion. The CTPS model revealed that there would be a 0.67 mph decrease in 24 h average speeds, accounting for congested periods during which the speed limit is irrelevant.

Outputs from traffic modeling helped us predict likely health outcomes associated with the proposed bill. Like all models, the CTPS traffic model made assumptions, interpolated, and extrapolated data. We sent a draft model for review by the 13 RPAs’ transportation planners and received feedback that we incorporated into the final model.

### 2.4. Pathways Linking Speed Limits and Health

**Figure 1:**
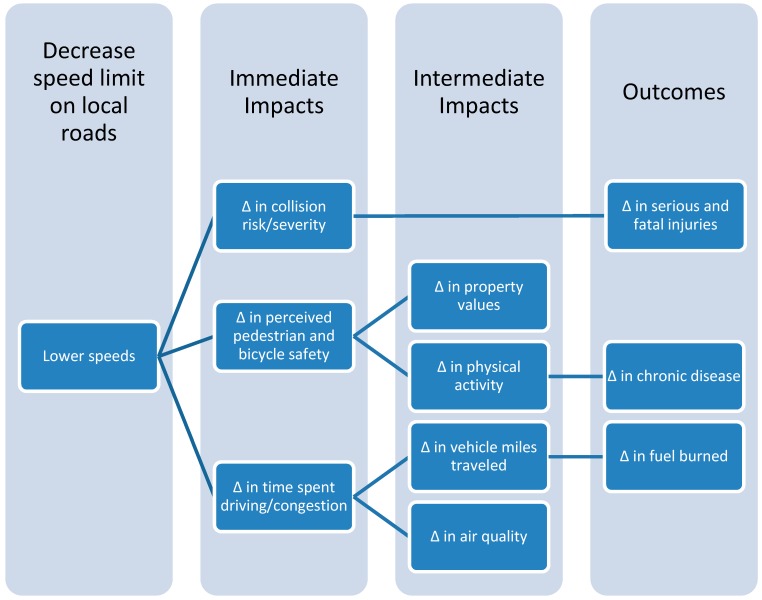
Pathways for the health impacts of speed limits.

As a result of a preliminary literature review and our stakeholder engagement process, we found that speed limit reductions could influence human health through multiple environmental, behavioral, and economic pathways, as shown in Figure 1. While data constraints prevented a complete quantitative analysis of impacts under all pathways, we were able to quantify effects associated with the following pathways linking speed limits to health:

Collisions, injuries, and fatalities
Fuel burned and time spent in traffic
Health effects of air pollution

While we could not quantify expected impacts, we estimated the likely direction and magnitude of effects of the bill on perceived pedestrian and cyclist safety and physical activity; and property values.

### 2.5. Assessment

#### 2.5.1. Collisions, Fatalities, and Injuries

Motor vehicle crashes account for a large share of the United States’ injury and mortality burden [1]. Because traffic speeds influence stopping distance, speed limits can reduce crash severity and traffic injuries [2].

*Methods for Assessment:* To estimate the effect of lowering speed limits on functionally classified local roads from 30 mph to 25 mph, we reviewed scientific literature to find an analytic approach that was both scientifically valid and feasible to implement in this rapid HIA. We selected a tool known as the “Power Model,” developed to describe the relationship between speed and road safety. It is named for a set of power functions relating changes in average traffic speeds to changes in the number and types of crashes and crash victims, including fatalities, fatal and serious injuries, and all injured road users [6]. Using predicted traffic speed reductions from the statewide transportation model described above, we used the Power Model to estimate how the number and severity of injuries and collisions would likely change if the Speed Limit Bill were passed. The model, based on 526 data points from 115 traffic studies, is parsimonious and conservative in predicting how changes in speed will affect changes in safety outcomes, and has been validated in numerous follow-up studies [2,6,7]. The model estimates how much a given decrease in speed will lower the risk of various types of crashes.

We collected baseline statewide crash data for 2006 through 2009, the most recent years for which data are available, from the Registry of Motor Vehicles (RMV) Crash Data System (CDS). The RMV Division of Massachusetts Department of Transportation (MassDOT) obtains crash reports from local and State police, as well as operators who were involved in crashes, and enters the data into CDS. Any crash involving an injury or fatality, or damage to any one vehicle or other personal property in excess of $1000 is reported, while those that are less severe and do not result in injury or substantial damage are left out of the system. It should be noted that crash statistics are usually underreported [8,9,10], but that in Massachusetts, 2009 data is of particularly poor quality. Beginning in 2009, the RMV did not have the resources to enter many of the crashes that were reported by vehicle operators. Due to changes in RMV reporting, 2009 data did not contain many of the crashes that presumably occurred on local roads.

We used geographic information systems (GIS) to map locations of crashes across MA from 2006–2009 to identify which took place on functionally classified local roads. This established baseline counts of total crashes, fatalities, injuries, pedestrian fatalities, pedestrian injuries, cyclist fatalities, and cyclist injuries specific to local roads. For each outcome, we used the Power Model to estimate the expected changes under the following speed reduction modeling assumptions: average speed declines by 1.8 mph, the change resulting from a 5 mph speed limit reduction based on estimates by Elvik *et al.* [2]. Average speed declines by 0.67 mph, the change resulting from a 5 mph speed limit reduction based on estimates by Elvik et al. [2] and taking into account congested traffic conditions as modeled by CTPS.

#### 2.5.2. Cost of Collisions

Deaths and injuries due to motor vehicle crashes have a tremendous economic impact: medical and work loss costs for deaths and emergency department-treated nonfatal injuries exceeded $90 billion across the United States in 2005 [11]. Lowering speed limits has the potential to decrease the frequency and severity of crashes, and this could also reduce their associated costs and the economic burden that they place on society.

*Methods for Assessment:* To estimate the economic impacts of preventing crashes through reduced speeds, we used the CDC Web-based Injury Statistics Query and Reporting System (WISQARS) Cost of Injury Reports application [12] with inputs from the Collisions, Fatalities, and Injuries section. The WISQARS database provides state-specific statistics on the costs associated with unintentional fatal and non-fatal injuries, including injury deaths, hospitalizations, and emergency department (ED)-treated (*i.e.*, treated in the ED but not hospitalized) cases by mechanism and intent of injury. WISQARS presents cost estimates in three mutually exclusive categories that reflect the severity of injury:

(1) injuries resulting in death, including deaths occurring within and outside a healthcare setting;
(2) injuries resulting in hospitalization with survival to discharge; and (3) injuries requiring an ED visit not resulting in hospitalization.

WISQARS estimates reflect total costs to society, which means they include all costs regardless of who pays for them. The application estimated medical and work loss costs of each death using 2005 National Vital Statistics System incidence data, while medical costs of non-fatal injuries primarily were derived from databases of the Healthcare Cost and Utilization Project. Medical costs for outpatient and physician office visits were not included, nor were non-medical and non-financial costs to society. Those include, but are not limited to, disability, mental/emotional anguish of surviving family member or co-workers, property damage, lowered property values, community fear, law enforcement, judicial, and litigation costs. As such, these are conservative estimates.

#### 2.5.3. Time Spent and Fuel Consumed in Traffic

Lowering speed limits has potential costs. Slower speeds on functionally classified local roads would result in more time behind the wheel for some individuals. In addition to more time driving, because cars often run at lower efficiencies at slower speeds there could be increases in fuel consumption.

*Methods for Assessment:* We obtained 2010 traffic data from CTPS meant to represent current conditions. We then commissioned CTPS to model the same parameters assuming that average speeds on local roads declined by 1.8 mph as a result of the Speed Limit Bill [13].

Estimates included vehicle-miles traveled (VMT) by automobiles and trucks, and vehicle-hours traveled (VHT) for each affected Transportation Analysis Zone (TAZ). Average fuel costs, vehicle occupancy, and monetary value of time were taken from a widely utilized annual publication, the Texas Transportation Institute (TTI) Urban Mobility Report [14].

Changes in VMT and VHT due to speed limit changes were estimated by CTPS. We multiplied the change in person-hours (VHT) by $16.94, or the value of one hour of travel time in the greater Boston region in 2012 USD assuming that 1.25 persons were in each car. We valued time spent driving in trucks at $91.60/h per vehicle, assuming trucks are used for commercial purposes, and assumed only one occupant per truck. Finally, we annualized these daily costs based on the CTPS-provided annualization factor of 300.

Fuel use under each scenario, in gallons, was calculated by using TTI equations below and the average speed on all road types to calculate average fuel economy in gallons per mile for trucks and automobiles separately [14]:

*Automobile Fuel Economy* = 0.0066 × (*speed*)^2^ + 0.823 × (*speed*) + 6.1577 *Truck Fuel Economy* = 1.4898 × ln(*speed*) – 0.2554

We then calculated the miles driven under each scenario by multiplying the VMT for automobiles and trucks using the commercial mix provided by CTPS. The miles driven for automobiles and trucks were then multiplied by the cost of fuel for each vehicle type, assuming that automobiles are fueled exclusively by gasoline and trucks are fueled exclusively by diesel, and using the Massachusetts Executive Office of Energy and Environmental Affairs (EEA) 2012 average gasoline cost of $3.48/gallon and an average diesel cost of $3.79/gallon [15].

#### 2.5.4. Air Pollution

Motor vehicles emit potentially harmful air pollutants, including particulate matter (PM), carbon dioxide, and nitrogen dioxide. Ozone can also form as secondary pollutant due to vehicle exhaust. Epidemiological evidence indicates air pollution contributes to mortality and hospitalizations due to asthma, chronic lung disease, heart attacks, ischemic heart disease, and major cardiovascular disease [16,17,18,19]. If reducing speed limits leads to more congested traffic, air pollution emissions would rise. Additionally, vehicles are typically designed to burn fuel most efficiently typically around 40–50 mph [20], and slower average traffic speeds would change the composition of vehicle emissions.

*Methods for Assessment:* We developed estimates of air pollution-related health impacts based on emissions models run by CTPS. CTPS used these emission data as inputs for MOBILE6.2, a vehicle emissions modeling software formerly used by the U.S. Environmental Protection Agency (EPA) [21]. Predicted county-level concentrations of fine particulate matter (PM_2.5_) were estimated using a Source-Receptor Matrix developed for the US EPA [22] for each speed limit reduction scenario. This matrix calculated how the emissions would change air pollution concentrations across Massachusetts, as well as in neighboring states.

We obtained county-level baseline data on hospitalization rates for asthma, chronic obstructive pulmonary disease (COPD), myocardial infarction (MI), and cardiovascular disease (CVD) from MassCHIP [23], and data on mortality rates from the US Centers for Disease Control and Prevention [24]. Health impacts due to air quality changes were calculated based on baseline rates and concentration-response relationships compiled in the EPA Environmental Benefits Mapping and Analysis Program (BenMAP) [25]—mortality [17,26], hospitalizations for asthma [18], CVD [16,27], MI [16], and COPD [28].

To monetize air pollution-related health impacts, we applied costs for deaths and hospitalizations from standard values used in U.S. EPA regulatory impact analyses [25,29]. The value of statistical life (VSL) of $8.32 million in 2012 USD was used to monetize mortality endpoints [30]. The values of a hospitalization event were taken from the U.S. EPA software BenMAP. The total value to society of an individual’s avoidance of a hospital admission can be thought of as having two components: (1) the cost of illness (COI) to society, which includes the total medical costs plus the value of the lost productivity, as well as (2) the willingness to pay (WTP) of the individual, as well as that of others, to avoid the pain and suffering resulting from the illness. However, BenMAP does not contain estimates of social WTP to avoid hospital admissions, and therefore estimates of total COI are conservative (lower bound) estimates.

#### 2.5.5. Pedestrian and Bicyclist Perceptions of Safety

Walking and bicycling for transportation helps people incorporate physical activity into everyday life, reducing the risk of many chronic diseases. A recent study by Lee et al. [31] estimates that physical inactivity causes 6% of the global burden of disease from coronary heart disease, 7% (range 3.9–9.6) of type 2 diabetes, and 9% (range 5.1–12.5) of premature mortality. Meeting the Surgeon General’s recommendation of 30 minutes of moderate intensity physical activity on most days of the week reduces risk of all-cause mortality, cardiovascular disease, and type 2 diabetes [32,33]. One way to increase the population’s rate of physical activity is by shifting the mode of transportation from automobiles to active modes, such as walking and bicycling. For example, a meta-analysis by Hamer and Chida [34] examined the association between commuting physical activity and cardiovascular risk and found that active commuting that incorporates walking and biking was associated with an 11% reduction in cardiovascular risk. One of the barriers, however, to facilitating this shift to active transportation may be negative perceptions of road safety due to excessive speeds of motorized vehicles.

*Methods for Assessment:* We conducted a rapid review of peer-reviewed literature on individuals’ perceptions of road safety as related to traffic speed and speed limit reductions, and on the health impacts of walking and bicycling. We used 2010 U.S. Census [35] and 2010 Behavioral Risk Factor Surveillance System (BRFSS) [36] data to describe current commuting patterns and levels of sedentary behavior and obesity in Massachusetts.

#### 2.5.6. Parental Safety Perceptions and Children’s Levels of Physical Activity

There is widespread recognition that childhood obesity and diseases related to a lack of physical activity among children, including pre-diabetes, diabetes, and asthma, are major public health challenges in the United States [37]. With a dramatic rise in childhood obesity rates occurring over the past several decades alone, researchers and policy makers have concluded that changes in environmental and contextual factors, rather than innate biological or genetic drivers, are likely to blame for the childhood obesity epidemic and may therefore be promising points of intervention [38,39,40]. One modifiable contextual risk factor that has gained considerable attention in recent years has been the built environment. National efforts are currently underway to help children become more physically active by improving the quality of the built environment for walking and biking.

Community-based interventions to encourage higher levels of physical activity among children via improvements to the built environment frequently focus on reducing traffic speed and volume. When successful “traffic calming” efforts reduce vehicle speeds and volumes and thereby prevent crashes and improve safety, they may also promote physical activity among children by favorably changing the built environment. Secondly, these changes may lead to increased perceptions of safety, causing parents and schools to encourage walking and biking among children, and increasing children’s willingness to walk and bike [41]. Not only can this benefit children by increasing their physical activity, it also may make them safer. Data indicates that the likelihood that a given person walking or bicycling will be struck by a motorist varies inversely with the number of individuals walking or bicycling [42]. Motorists appear to adjust their behavior in the presence of people walking and bicycling.

*Methods for Assessment*: We summarized the literature on whether lower traffic speeds translate to higher levels of perceived safety among parents or children, increased willingness to allow children to play walk or bike outside, and ultimately levels of childhood physical activity.

#### 2.5.7. Property Values

As well-recognized “social determinants of health,” socioeconomic factors are known to influence whether people get sick or stay healthy [43]. Socioeconomic factors can influence behaviors, enforce norms, and reduce or buffer stress [44]. This section examines whether property values, a component of neighborhood socioeconomic status, could be affected by speed limit reductions, in turn impacting health via changes in homeowner wealth or local housing conditions.

Traffic speeds on roads predict adjacent property values, as homebuyers pay a premium for quieter, safer streets. In a survey of homebuyer preferences, community design with low traffic ranked as the top priority out of 39 attributes used to select a home [45]. A 5 to 10 mph reduction in traffic speeds is associated with an increase in nearby residential property values by approximately 2% [46]. Other studies have demonstrated that reducing the volume of traffic on residential streets can increase property values [47,48,49]. Reducing traffic speeds therefore has the potential to impact residential property values for homes across the state.

*Methods for Assessment:* We reviewed the literature on the relationship between household values and traffic speeds and then reviewed 2006–2010 American Community Survey (ACS) from the US Census for estimates on median home values in Massachusetts [50].

## 3. Results and Discussion

### 3.1. Results

#### 3.1.1. Collisions, Fatalities, and Injuries

Local roads make up about 52% (18,945) of Massachusetts’ 36,247 miles of roads (MassDOT 2012). Table 1 shows crash data for Massachusetts. From 2006-2009, there were 477,103 total collisions reported by the RMV. Twenty-nine percent of these collisions (136,262) occurred on “functionally classified local roads” that would have been impacted by the Speed Limit Bill. Of total collisions, 23% (314) of fatal injuries and 26% (44,580) of nonfatal injuries occurred on these local roads from 2006–2009. Approximately 37% of crashes involving pedestrians and crashes involving cyclists occurred on local roads (Table 2 and Table 3).

**Table 1:** Motor vehicle crashes from 2006–2009 reported by the registry of motor vehicles.

Crash Type	2006	2007	2008	2009	Total	Annual Average
Crashes on All Roads	124,274	120,667	120,970	111,192	477,103	119,276
Crashes on Local Roads	34,832	34,953	34,319	32,158	136,262	34,066
Fatal Crashes on All Roads	384	400	321	292	1397	349
Fatal Crashes on Local Roads	97	93	69	55	314	79
Fatalities on All Roads	410	426	345	316	1,497	374
Fatalities on Local Roads	101	100	71	59	331	83
Injury Crashes on All Roads	33,038	31,289	31,017	28,933	124,277	31,069
Injury Crashes on Local Roads	8543	8196	7845	7560	32,144	8,036
Injuries on All Roads	45,934	42,947	42,321	40,077	171,279	42,820
Injuries on Local Roads	11,991	11,334	10,741	10,514	44,580	11,145

**Table 2:** Pedestrian crashes from 2006–2009 reported by the registry of motor vehicles.

Crash Type	2006	2007	2008	2009	Total	Annual Average
Crashes on All Roads	124,274	120,667	120,970	111,192	477,103	119,276
Crashes on Local Roads	34,832	34,953	34,319	32,158	136,262	34,066
Crashes Involving Pedestrians on All Roads	1577	1584	1765	1671	6597	1649
Crashes Involving Pedestrians on Local Roads	576	585	672	615	2,448	612
Pedestrian Fatalities on All Roads	58	65	72	46	241	60
Pedestrian Fatalities on Local Roads	19	15	28	13	75	19
Pedestrian Injuries on All Roads	1116	1192	1316	1331	4955	1239
Pedestrian Injuries on Local Roads	408	437	478	479	1802	451

**Table 3:** Cyclist crashes from 2006-2009 reported by the registry of motor vehicles.

Crash Type	2006	2007	2008	2009	Total	Annual Average
Crashes on All Roads	124,274	120,667	120,970	111,192	477,103	119,276
Crashes on Local Roads	34,832	34,953	34,319	32,158	136,262	34,066
Crashes Involving Cyclists on All Roads	1069	1069	1227	1248	4613	1153
Crashes Involving Cyclists on Local Roads	398	393	458	455	1704	426
Cyclist Fatalities on All Roads	6	11	10	6	33	8
Cyclist Fatalities on Local Roads	1	8	4	1	14	4
Cyclist Injuries on All Roads	753	744	866	882	3245	811
Cyclist Injuries on Local Roads	277	281	309	313	1180	295

The second scenario conservatively assumed that serious and fatal crashes are just as likely to take place in congestion as they are in free flow traffic conditions and therefore modeled changes due to the 24 h average speed reduction, which accounted for congestion. Estimates that assume traffic on local roads would slow by 1.8 mph, on average, in response to a 5 mph lower speed limit, show that the Speed Limit Bill could have prevented roughly 2200 crashes, 18 fatalities, and 1200 injuries per year across the Commonwealth.

Table 4 shows expected annual decreases in crashes, injuries, and fatalities under the two speed reduction modeling assumptions. The first modeling assumption scenario assumed that crashes serious enough to cause injury or fatality, or damage to any one vehicle or other personal property in excess of $1000 were unlikely to take place in congested conditions, and therefore modeled the impact of the bill without accounting for congestion.

**Table 4:** Estimated annual reductions in crashes and fatalities (power model results).

Estimated Annual Decrease in	1.8 mph Speed Reduction Estimate (95% Confidence Interval)	0.67 mph Speed Reduction Estimate (95% Confidence Interval)
Total Crashes	2219 (286, 4042)	811 (102, 1505)
Fatal Crashes	15 (2, 27)	6 (1, 11)
Injury Crashes	772 (460, 1072)	285 (168, 401)
Fatalities	18 (−4, 35)	7 (−1, 15)
Injured Road Users	1239 (369, 2039)	460 (133, 77)
Pedestrian Fatalities	4 (−1, 8)	2 (0, 3)
Cyclist Fatalities	1 (0, 1)	0.3 (−0.1, 0.6)
Injured Pedestrians	50 (15, 82)	19 (5, 31)
Injured Cyclists	33 (10, 54)	12 (4, 21)

Note: These numbers should not be summed across types of crashes/health outcomes. Some categories are subsets of other categories

Estimates that consider congestion would slow traffic by 0.67 mph on average and could prevent roughly 810 crashes, seven fatalities, and 460 injuries per year across the Commonwealth. It should be noted that the figures for fatalities were not statistically significant, although point estimates suggest a protective effect of lower speeds. There is consistent evidence that reducing traffic speeds decreases the frequency and severity of crashes. Despite the predicted magnitude of these benefits, there are a number of limitations to this analysis. The Power Model, while parsimonious, is fairly crude. Like any model, it makes assumptions and is not completely precise and accurate. Additionally, while speeds decrease and safety increases on local roads, the CTPS model demonstrates that traffic volume may increase on highways and arterials. It should be noted, however, that average speeds on highways and arterials will decrease slightly due to increased congestion, potentially increasing safety on these roads as well. Finally, our estimates are based on imperfect data. As stated earlier, crash data is consistently underreported. As such, the estimates presented here are conservative, and more accurate data would reveal greater decreases in crashes, injuries, and fatalities.

#### 3.1.2. Cost of Collisions

Recent CDC estimates show that the cost of death from motor vehicle crashes in Massachusetts was $394 million in 2005 (Centers for Disease Control and Prevention 2011). Work loss costs made up $388 million of these costs, while medical costs made up $6 million. Table 5 and Table 6 show the estimates of how much preventing injuries and fatalities based on a 1.8 mph and 0.67 mph decrease in traffic speeds (from the Power Model) could save in terms of both work loss and medical costs. Under both sets of assumptions, a speed limit reduction of 5 mph would result in savings. Estimated savings ranged from $11–$30 million in prevented fatalities and between $67–$180 million in prevented injuries. These savings would affect those involved in collisions and their families, as well as employers, property owners, and taxpayers across the state. Limitations to this analysis include the lack of data on costs for collisions that did not result in an injury or fatality. Including these personal damage costs would increase cost savings estimates. This analysis assumes that all injuries prevented by the modeled reduced speeds would have otherwise resulted in a hospital visit. This assumption is based on the fact that our baseline data came from the RMV CDS, which only registers serious crashes.

**Table 5:** Estimated costs savings for a 1.8 mph decrease in traffic speeds based on CDC’s WISQARS in 2012 dollars.

**Outcome**	**Fatalities**	**Pedestrian Fatalities**	**Cyclist Fatalities**
Annual Decrease in Deaths	18	4	1
Medical Cost Avoided	$346,721	$76,699	$18,912
Work Loss Cost Avoided	$29,347,334	$6,521,513	$1,630,641
Combined Cost Savings	$29,694,055	$6,598,212	$1,649,553
**Outcome**	**Injured Road Users**	**Injured Pedestrians**	**Injured Cyclists**
Annual Decrease in Number Hospitalized	1239	50	33
Medical Cost Avoided	$63,872,373	$2,703,376	$1,652,705
Work Loss Cost Avoided	$116,610,789	$5,164,047	$3,766,654
Combined Cost Savings	$180,483,163	$7,867,423	$5,419,359

**Table 6:** Estimated costs savings for a 0.67 mph decrease in traffic speeds based on CDC’s WISQARS in 2012 dollars.

**Outcome**	**Fatalities**	**Pedestrian Fatalities**	**Cyclist Fatalities**
Annual Decrease in Deaths	7	2	0
Medical Cost Avoided	$133,435	$37,824	$0
Work Loss Cost Avoided	$10,990,016	$3,140,455	$0
Combined Cost Savings	$11,123,451	$3,178,279	$0
**Outcome**	**Injured Road Users**	**Injured Pedestrians**	**Injured Cyclists**
Annual Decrease in Number Hospitalized	460	19	12
Medical Cost Avoided	$23,713,638	$1,027,556	$600,984
Work Loss Cost Avoided	$43,293,937	$1,962,653	$1,370,075
Combined Cost Savings	$67,007,575	$2,990,209	$1,971,058

#### 3.1.3. Time Spent and Fuel Consumed in Traffic

Base year conditions from the CTPS model indicated that the total daily VMT for the state were 155.1 million, of which 26.6 million were on local roads, making up 17% of total daily VMT. The total daily VHT were 4.6 million. The CTPS model showed that under the 1.8 mph reduction in traffic speeds, daily VMT on total roads would increase by 184,000, while daily VHT on total roads would increase by 19,000. Daily VMT overall would increase as drivers choose new, less direct routes to avoid slower traffic on local roads. Daily VMT on local roads would decrease by 355,676, while daily VHT on local roads would increase by 2860 as a result of the bill.

Applying the TTI equations to these data, we found that fuel costs would increase by $21 million per year and the increased time spent in traffic would cost Massachusetts drivers $127 million in lost time. CTPS also modeled whether participants would shift from commuting by automobile to biking, walking, or public transit as a result of the speed reductions, and found that there would be no appreciable mode shifts.

While speed limit reductions would reduce crashes and prevent injuries and fatalities, they would also prompt drivers to reduce cut-through traffic by seeking faster, though often longer distance, routes on higher capacity roads, resulting in an additional 55.3 million vehicle miles travelled per year. At the same time, slower travel speeds on local roads and higher traffic volumes on newly preferred, higher capacity roads would result in 5.8 million additional VHT per year. These increases in time spent in traffic would cost approximately $127 million per year, while increases in fuel consumed in traffic would cost $21 million per year.

#### 3.1.4. Air Pollution

In general, most monitored air pollutants in the state of Massachusetts are at levels below health-based standards, and levels have been declining over time [51]. Estimates of changes in air pollutants under both speed reduction scenarios would be minor, but concentrations would lead to slight increases in risk for the citizens of Massachusetts. A speed limit reduction was expected to increase traffic congestion and related air pollution emissions, prompting us to investigate the potentially harmful health effects of additional air pollution associated with the Speed Limit Bill. Although higher concentrations of air pollutants would contribute to additional deaths and hospitalizations due to asthma, chronic lung disease, heart attacks, ischemic heart disease, and major cardiovascular disease, models showed that modeled increases associated with the bill would be negligible. The total annual expected number of increased illnesses due to the increase in air pollution was lower than 0.001 cases per year for all outcomes. Air pollution-related health costs would be approximately $474 per year.

Final estimates do not include the effects of exposure to other pollutants that may change as an impact of the bill, including SO_2_, CO, ozone, and ultrafine particles. We relied upon air pollution estimates from CTPS that use the EPA’s MOBILE6.2 model, which does not incorporate additional emissions that would occur due to stop-and-go traffic. Additionally, we were not able to calculate effects of air pollution on stroke, premature birth, infant mortality, and childhood asthma. These factors would contribute additional mortality and hospitalizations not calculated here. These aggregated numbers do not demonstrate the distribution of risk among different populations. Finally, our estimates also do not include increased exposures specific to commuters, who may spend more time in traffic in close proximity to elevated concentrations.

#### 3.1.5. Pedestrian and Bicyclist Perceptions of Safety

According to the 2010 Census, nearly 200,000 (6%) Massachusetts workers bike or walk to work, however about 20% of Massachusetts residents report engaging in no leisure time physical activity [35]. Although Massachusetts is considered one of the healthiest states in the country, 60% of adults are overweight and 24% of adults are obese, highlighting the significance of interventions that help residents become more active [36].

While traffic calming strategies are primarily promoted as a way to reduce crashes, injuries and deaths, they may also be a feasible method to promote physical activity by helping create an environment that encourages active transportation. The World Health Organization has suggested that traffic may have a strong negative impact on health by reducing the ability to engage in active transportation [52]. One pathway that the negative impact of traffic may have on physical activity is through the perception of safety. Studies that consider traffic and perceptions of safety generally agree that pedestrians and bicyclists have negative perceptions of traffic and that real and/or perceived danger and discomfort in traffic discourages walking and bicycling [41,42,53,54,55,56,57,58]. Safety concerns appear to be strongest in children, the elderly and women, thus contributing to health inequalities for these groups [56,59].

Evidence generally supports the positive impact of traffic calming overall on perceptions of safety and active transportation. Speed limit reductions would likely create more conducive conditions for active transportation by improving the objective safety of roads for all users.

#### 3.1.6. Parental Safety Perceptions and Children’s Levels of Physical Activity

In the United States, almost half of elementary and middle school students walked or biked to school in 1969, whereas less than 15% walk or bike to school today [42]. Almost 17% of children aged 2 to 5 and 11% of middle school students are overweight in Massachusetts [60].

The weight of the evidence reviewed indicates that higher traffic speed/density is associated with lower levels of physical activity among youth [61]. Conversely, classic traffic calming measures, such as controlled intersections, and supportive infrastructure, such as sidewalks, were associated with higher levels of physical activity. Additionally, Morrison and colleagues [41] report an increase in parents’ willingness to allow children to walk and ride bicycles after the implementation of a traffic calming scheme. Objective measures of traffic indicated that safer pedestrian environments (e.g., slower speeds and lower traffic volume) predicted higher levels of physical activity. Carver and colleagues note that traffic-calming measures, quiet local streets with a speed limit of 50 km/h or less (about 31 mph), and higher street connectivity had the most positive impact on physical activity behaviors and active transportation [62]. It is likely that the Speed Limit Bill would have supported more physical activity among children across the Commonwealth; however, accompanying bicycle/pedestrian facilities and other self-enforcing engineering interventions would maximize perceived safety.

#### 3.1.7. Property Values

American Community Survey Data from the US Census Bureau show that the average Census tract median home value for the state of Massachusetts for 2006–2010 was $374,499 [50]. According to a recent analysis of tax data conducted by the Boston Globe, statewide home values have more than doubled since 2000; however, home values hit an all-time high in 2007 and have dropped since the 2008 recession.

We find a consistent relationship between lower traffic speeds and higher property values [45,46,47,48,49]. The literature, however, is sparse and could not be reliably extrapolated to assess the likely impact of the Speed Limit Bill on property values in Massachusetts, especially because all properties on local roads would be affected simultaneously. However, the literature indicates general preferences for the safety and quiet associated with slower speeds, suggesting that residents would likely enjoy quality of life benefits even if they were not monetized into higher home values.

#### 3.1.8. Results Summary

Based on a literature review, case studies, and statistical models, the HIA predicted that lowering speed limits on local roads would have had a positive public health impact across Massachusetts, particularly by preventing traffic fatalities and injuries. Potential co-benefits included enhanced walking and biking environments that would encourage physical activity, as well as increased desirability of properties on local roads due to quieter and safer streets. The HIA also concluded that the bill is economical. The Speed Limit Bill would have prevented 2219 crashes per year, 18 fatalities per year, and 1239 injuries per year, which translates into a savings of up to $210 million annually in prevented medical payments and work lost. These economic benefits outweighed the costs of increased time spent in traffic and fuel burned estimated in our models, as well as the health impacts of the small increase in air pollution, associated with the proposed change. In addition, lower speed limits would have likely improved children’s and adults’ perceptions of road safety, which could have led to increased pedestrian and bicyclist physical activity and a resulting reduction in chronic disease risk. Although the evidence was limited, we predicted that Massachusetts residents living on local roads would have experienced increased satisfaction with their neighborhoods as a result of the proposed bill even if these benefits were not monetized.

### 3.2. Recommendations

Our primary recommendation was that the legislature should adopt a bill to lower speed limits on local roads. To maximize the effectiveness of a speed limit reduction, we suggest combining a change in the law with the following evidence-based recommendations.

#### 3.2.1. Further reductions in Motor Vehicle Speeds

Further measures to decrease traffic speeds in conjunction with lower speed limits include traffic calming, enforcement, and education. To explore the potential impact of further reductions in traffic speeds to the posted 25 mph limit, we modeled the impact of a full 5 mph decrease on crashes and fatalities on functionally classified local roads.

Our analysis suggests that a 5 mph reduction in traffic speeds would confer three times the reduction in crashes and twice the cost savings of a 1.8 mph reduction. Most importantly, the 5 mph decrease would save more than twice the number of lives compared to a 1.8 mph reduction (Table 7).

**Table 7:** Crashes and Cost of Crashes in 2012 dollars.

Estimated Annual Decrease in:	1.8 mph Speed Reduction	5 mph Speed Reduction
**Total Crashes**	2219 (95% CI 286, 4042)	6265 (95% CI 855, 10,794)
**Fatalities**	18 (95% CI −4, 35)	44 (95% CI −11, 67)
**Injured Road Users**	1239 (95% CI 369, 2039)	3336 (95% CI 1077, 5088)
**Medical and Work Lost Cost of Fatalities**	$29,694,055	$72,586,636
**Medical and Work Lost Cost of Hospitalizations**	$180,483,163	$485,949,909

Given these findings, measures to help align true traffic speeds with the regulated speed would have significant health and economic benefits. In conjunction with a speed limit reduction, traffic-calming design solutions, plus enforcement and education, may help maximize health benefits associated with reduced traffic speeds.

#### 3.2.2. Implementation—Dissemination

We recommended that the Speed Limit Bill should be accompanied by a public information campaign to help the public understand the bill’s costs and benefits. The campaign could include a media component, inclusion in the driver’s education curriculum, inclusion in RMV mailings or other documents regularly distributed to drivers.

#### 3.2.3. Implementation—Enforcement

Enforcement policies and policing would help reduce actual traffic speeds closer to the 25 mph limit. Speed cameras may prevent speeding and crashes [63], and enforcement approaches that remind drivers that roads are patrolled for speeding may also help raise compliance rates with lower speeds.

#### 3.2.4. Implementation—Traffic Calming

To maximize the health benefits of speed reductions, passive and self-enforcing engineering interventions are most effective [64]. New local roads designed to support a lower speed limit would be most beneficial to health. If the road design speed differs from the speed limit on existing roads, traffic calming measures could help reduce travel speeds without intensive enforcement. Traffic calming is an engineering strategy that slows traffic and reduces traffic volume [65]. Studies show that traffic calming measures can reduce road traffic injuries by roughly 15% in the areas that received design and engineering interventions [66].

Measures that change the height of the road surface appear to be among the most effective in reducing speeds and preventing injuries and fatalities [64]. “Vertical deflections”, such as raised pedestrian crossings, speed humps, and cushions, alter the road surface height and force drivers to slow. Less effective approaches narrow roadways and/or create “horizontal deflections” (e.g., pinch-points, bump-outs, roundabouts, islands, chicanes,) that force vehicles to veer, and therefore slow.

Municipalities should also consider implementing traffic calming interventions that serve pedestrians and cyclists, including raised crosswalks, reducing motor vehicle lane width to serve bicycles, and signalization to accommodate active road users [57,67,68].

### 3.3. Dissemination and Impact Evaluation

In January 2013, MAPC, in collaboration with WalkAmerica, WalkBoston, MDPH, and MassDOT, convened stakeholders and traffic safety experts for a “Slow Down Summit”. The Summit allowed attendees to participate in discussions about strategies to slow traffic down and improve bicyclist and pedestrian safety. The theme was safe speeds for vibrant communities, and was standing room only. The summit allowed us to disseminate our preliminary findings, receive feedback, and engage policymakers on the topic of speed limits and their health impacts.

**Figure 2:**
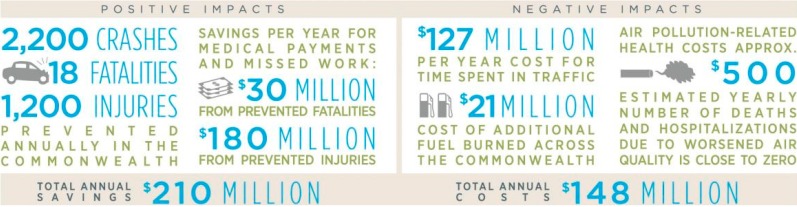
Infographic of estimated total savings and costs under the Speed Limit Bill.

The HIA was released via press release on 12 September 2013. There was a delay in the release of the HIA because the Speed Limit Bill did not pass during the 2011–2012 legislative session. The bill was re-filed in the next session, however the new bill did not define roads by functional classification. Therefore, the HIA did not apply to the new bill as filed. However, the HIA aided in providing a balanced quantitative and qualitative analysis to show that reducing vehicle speeds on local roads has a net benefit for health and costs to society (Figure 2), and may inform future legislative efforts to improve traffic safety in Massachusetts.

Although the bill did not pass, the HIA process brought health more prominently into what had traditionally been viewed as a transportation discussion. Its quantitative and qualitative approach may be a useful model for transportation and public health professionals seeking to analyze how driving speeds impact road safety and other health risk factors. The HIA demonstrates an approach for assessing the health impacts of speed-related policy changes that can be easily borrowed by other HIA practitioners and public agencies. Finally, the HIA serves to increase awareness of the multiple benefits of slower traffic speeds.

## 4. Conclusions

Reducing speed limits on local roads would have protected health by making the roads safer for all users of the road. However, speed limit reductions alone would not reduce speeds to the regulated limits; enforcement and road engineering would also be needed to slow speeds. While a lower speed would increase congestion, VMT, and worsen air quality, the benefits outweigh the costs from both a health and economic perspective. Lowering speed limits on local roads could additionally be the catalyst for promoting alternative modes of transportation. To maximize the impact of lowering speed limits, state and local municipalities must work together to enforce policies and engineer roads that reflect the desired speed of a road and simultaneously make concrete efforts to promote alternative modes of transportation, such as walking or biking.
